#  Frequent HIV and Young Age Among Individuals With Diverse Cancers at a National Teaching Hospital in Malawi

**DOI:** 10.1200/JGO.17.00174

**Published:** 2018-06-08

**Authors:** Marie-Josèphe Horner, Ande Salima, Chrissie Chilima, Matthews Mukatipa, Wiza Kumwenda, Coxcilly Kampani, Fred Chimzimu, Bal Mukunda, Tamiwe Tomoka, Maurice Mulenga, Richard Nyasosela, Steady Chasimpha, Charles Dzamalala, Satish Gopal

**Affiliations:** **Marie-Josèphe Horner** and **Satish Gopal,** University of North Carolina at Chapel Hill, Chapel Hill, NC; **Marie-Josèphe Horner**, **Ande Salima**, **Chrissie Chilima**, **Matthews Mukatipa**, **Wiza Kumwenda**, **Coxcilly Kampani**, **Fred Chimzimu**, **Bal Mukunda**, **Tamiwe Tomoka**, **Maurice Mulenga**, **Richard Nyasosela**, and **Satish Gopal**, University of North Carolina Project-Malawi; **Marie-Josèphe Horner**, **Ande Salima**, **Chrissie Chilima**, **Matthews Mukatipa**, **Wiza Kumwenda**, and **Satish Gopal**, Kamuzu Central Hospital Cancer Registry; **Satish Gopal**, Malawi Cancer Consortium & Regional Center of Research Excellence for Non-Communicable Diseases, Lilongwe; **Steady Chasimpha** and **Charles Dzamalala**, Malawi Cancer Registry; and **Charles Dzamalala** and **Satish Gopal**, University of Malawi College of Medicine, Blantyre, Malawi.

## Abstract

**Purpose:**

Cancer surveillance provides a critical evidence base to guide cancer control efforts, yet population-based coverage in Africa is sparse. Hospital-based registries may help fill this need by providing local epidemiologic data to guide policy and forecast local health care needs. We report the epidemiology of patients with cancer recorded by a de novo hospital-based cancer registry at Kamuzu Central Hospital, Malawi, the sole provider of comprehensive oncology services for half the country and location of a high-volume pathology laboratory.

**Methods:**

We conducted active case finding across all hospital departments and the pathology laboratory from June 2014 to March 2016. Patient demographics, tumor characteristics, treatment, and HIV status were collected. We describe epidemiology of the cancer caseload, registry design, and costs associated with registry operations.

**Results:**

Among 1,446 registered patients, Kaposi sarcoma and cervical cancer were the most common cancers among men and women, respectively. Burkitt lymphoma was most common cancer among children. The current rate of pathology confirmation is 65%, a vast improvement in the diagnostic capacity for cancer through the hospital’s pathology laboratory. Among leading cancer types, an alarming proportion occurred at young ages; 50% of Kaposi sarcoma and 25% of esophageal, breast, and cervical cancers were diagnosed among those younger than 40 years of age. A systematic, cross-sectional assessment of HIV status reveals a prevalence of 58% among adults and 18% among children.

**Conclusion:**

We report a high caseload among typically young patients and a significant burden of HIV infection among patients with cancer. In low- and middle-income countries with intermittent, sparse, or nonexistent cancer surveillance, hospital-based cancer registries can provide important local epidemiologic data while efforts to expand population-based registration continue.

## INTRODUCTION

Malawi is experiencing a rapidly increasing cancer burden.^1^ Cancer incidence in Malawi is predicted to increase by 4% per year through 2030 due to demographic shifts, and the majority of new cases are anticipated to occur among those younger than 65 years of age.^1^ Malawi is simultaneously tackling a generalized HIV epidemic, with HIV seroprevalence now 9%.^2^ Reflecting the regional HIV epidemic in sub-Saharan Africa, two AIDS-defining cancers, Kaposi sarcoma and cervical cancer, are the most common malignancies among men and women, respectively, nationwide.^3^ In view of this dual burden of disease, high-quality, descriptive epidemiologic data are critical for Malawi to develop a national cancer control plan.

Cancer surveillance provides a critical evidence base to guide cancer control efforts and health policy. However, sub-Saharan Africa has large gaps in population-based cancer registries because of scarce or nonexistent funding, limited infrastructure, and poor diagnostic capacity in many regions. The African Cancer Registry Network has 25 member population-based registries across 18 sub-Saharan countries and a population coverage of 14% for the continent.^4^ Only 1% of Africa’s population is covered by five population-based cancer registries that meet the International Agency for Research in Cancer data quality standards of completeness, validity, and timeliness in reporting.^4,5^ In settings where coverage is absent, hospital-based registries may partially address these gaps by providing local descriptive epidemiologic data.^6,7^

We therefore established a hospital-based cancer registry with the aim of measuring and describing cancer caseload at Kamuzu Central Hospital (KCH). KCH is one of two national teaching hospitals in Malawi and home to a region-leading pathology laboratory.^8^ Located in the capital city, Lilongwe, KCH provides pathology and oncology services to a referral base of approximately 8 to 9 million residents throughout Malawi’s northern and central regions. In this article, we describe epidemiology characteristics of patients with cancer, design of the registry, and future directions for cancer surveillance in Malawi.

## METHODS

### Cancer Registration

The KCH Cancer Registry collects information on demographics, tumor characteristics, basis of diagnosis, HIV status, and basic treatment information for all persons with cancer presenting to the hospital (Table 1). Precursor conditions such as cervical dysplasia were excluded. The chosen data elements were prioritized on the basis of frameworks for cancer registration and recommendations in low-resource settings.^6,9,10^ Feasibility of data collection was based on a quantitative assessment of archived cancer diagnoses collected at KCH by the University of North Carolina from September 2009 to April 2014.

**Table 1:**
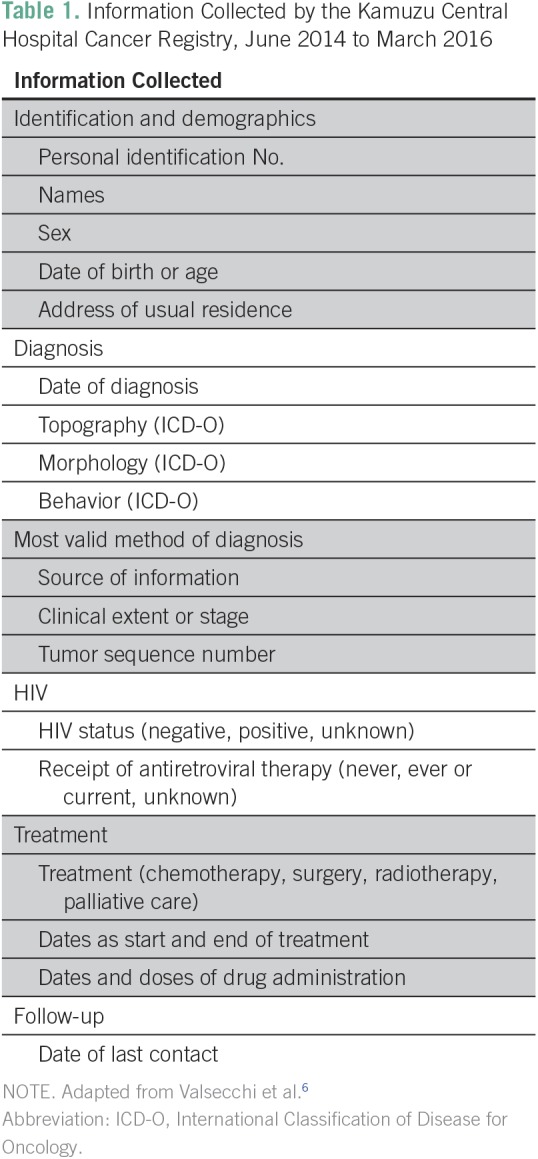
Information Collected by the Kamuzu Central Hospital Cancer Registry, June 2014 to March 2016

Since May 2014, two data clerks performed active case finding on a weekly basis across all hospital wards, the pathology laboratory, and the Lighthouse Trust HIV clinic, located on the KCH campus. Sources of information for case abstraction included medical charts, hospital ward logbooks, admission records from the Lighthouse Trust HIV clinic, electronic pathology records, and patient interviews, when possible. The Malawi health passport, a portable abridged medical record belonging to the patient, supplemented information abstracted from oncology clinic medical charts when it was available. Characteristics and basis of diagnosis were categorized according to SEER program coding.^11^ Treatment modality, defined as chemotherapy, surgery, palliative care, hormonal therapy, and radiotherapy, was piloted to assess feasibility of data abstraction. Treatment information was abstracted primarily through medical charts, when these were available. Electronic pathology records were manually parsed into cancer topography and histologic type (M.-J.H., C.C., A.S.). Data were coded using the International Classification of Disease for Oncology.^12^ Hospital death certificates rarely contributed to case ascertainment because they were seldom issued or available. Data abstraction occurred along the continuum of health care delivery, from initial diagnosis through treatment.

Synonyms for invasive cancers, case-finding terms, and ambiguous terminology^11^ were compiled into a case-finding reference dictionary that was used during data collection. Case abstraction used standardized data collection forms. Double data entry was conducted in a Microsoft Access 2010 frontend to an SQL Server data base (Microsoft, Redmond, WA), maintained locally in Lilongwe. Data were archived weekly at the University of North Carolina, Chapel Hill, North Carolina.

Quality control was conducted quarterly to evaluate missing data and major and minor errors. An independent data clerk conducted quality control of data coding and entry (M.M.). Periodic audits by a cancer epidemiologist (M.-J.H.) were conducted to assess the completeness of cases abstracted from pathology reports. Deduplication of records was routinely conducted by registry staff (C.C., A.S., M.M., W.K.).

### Analysis

To characterize overall cancer burden at KCH, descriptive statistics were generated for patient demographics, tumor characteristics, HIV status, treatment, and sources of information from June 2014 to March 2016. Tumor types were categorized into larger groups for analysis. Separate analyses were conducted for adults ≥ 20 years of age and children and adolescents ages 0 to 19 years. Pediatric patients were grouped and analyzed according to the International Classification of Childhood Cancer (3rd edition), which emphasizes morphology rather than primary site.^13^ The crude cost per case abstracted was estimated for comparison with other cancer registries in resource-limited settings. Cost per case in US dollars was calculated as direct and indirect total registry operating costs divided by the number of incident cases diagnosed over the study period. Descriptive analyses were generated using SAS/STAT software, Version 9.4, of the SAS system for Windows (SAS Institute, Cary, NC).

## RESULTS

### Information Sources and Costs

Among 1,446 cancers recorded from June 2014 to March 2016, 67% were registered at the time of initial cancer diagnosis, 32% during treatment or follow-up, and < 1% through hospital-reported deaths (Table 2). Laboratory diagnoses were a primary source of information for 47% of records during case abstraction. Case ascertainment was conducted across all major hospital departments, with the majority of cases recorded from the oncology clinic (26%) and pathology laboratory (33%). As expected, KCH receives a large number of oncology referrals from surrounding districts; 20% of patients reported their usual residence as outside of the Lilongwe district. The estimated total cost per case abstracted was US$20.10. Indirect costs accounted for 8% of total operations. Direct costs included costs associated with active case finding throughout all hospital departments, quality control, and data base maintenance.

**Table 2:**
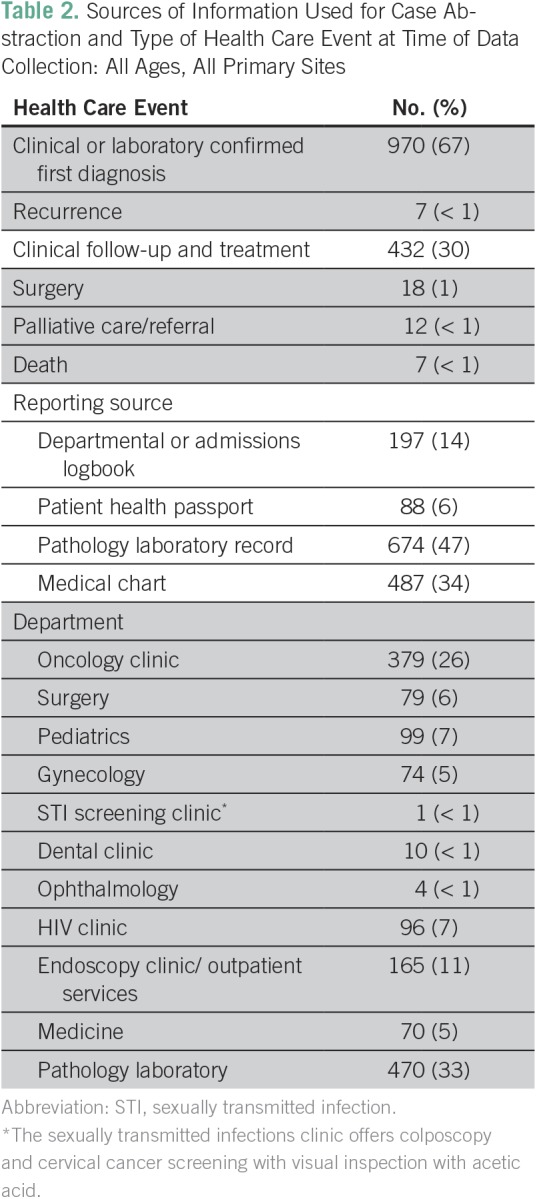
Sources of Information Used for Case Abstraction and Type of Health Care Event at Time of Data Collection: All Ages, All Primary Sites

### Adults

**Fig 1:**
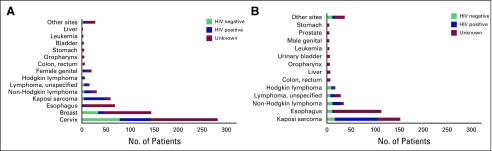
Distribution of patients with cancer and HIV status among men and women: (A) female and (B) male. Other sites include (A and B) anus; bones and joints; larynx; other digestive sites; skin, including melanoma; soft tissue; unspecified primary site; and (B) male breast.

**Fig 2:**
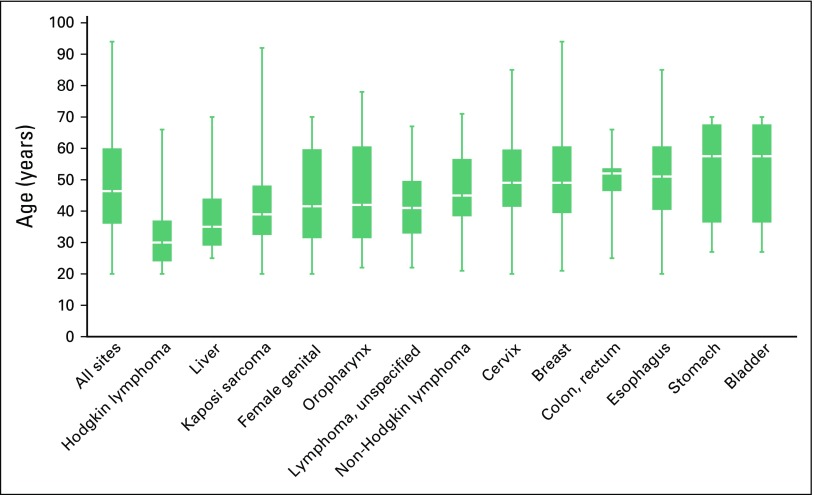
Median age at cancer diagnosis among adults ≥ 20 years of age by primary tumor site (median, interquartile range).

A total of 1,104 malignancies were recorded among adults (Figs 1A and 1B). Women represented 62% of patients. The most common cancers among women were cervix (42%), breast (21%), esophagus (10%), and Kaposi sarcoma (9%; Fig 2A). Among men, the most common cancers were Kaposi sarcoma (35%), esophagus (26%), and Non-Hodgkin lymphoma (8%; Fig 2B).

Among 491 patients with documented HIV status, overall HIV prevalence was 58%, which varied across cancer sites. Among women with known HIV status, the prevalence was 92% among patients with Kaposi sarcoma, 45% among those with cervical cancer, and 24% among those with breast cancer (Fig 2A). Among men, HIV prevalence was 84% among patients with Kaposi sarcoma and 41% among patients with non-Hodgkin lymphoma (Fig 2B). Receipt of antiretroviral therapy was unknown for 62% of patients with cancer coinfected with HIV, whereas 38% had a record of current or prior use.

Median age at diagnosis for all sites combined was 46 years (Fig 2). Among all leading cancer sites, a significant proportion occurred at ages older than 40 years. The median age at diagnosis for Kaposi sarcoma was 40 years. Among patients with esophageal cancers, 25% were diagnosed among those younger than 40 years of age (median age at diagnosis, 51 years). Among patients with lymphoma, the median age at diagnosis was 30 years for Hodgkin lymphoma, 47 years for non-Hodgkin lymphoma, and 45 years for other lymphomas of unspecified histology. Among women, 25% of patients with breast and cervical cancer were diagnosed before 40 years of age (median age at diagnosis, 49 years).

**Fig 3:**
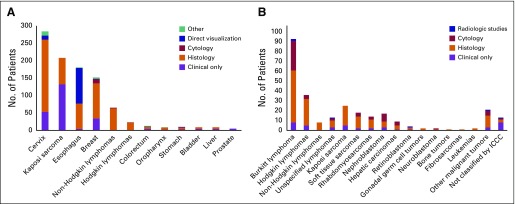
Mode of cancer diagnosis among (A) adults and (B) children and adolescents. Direct visualization without pathology confirmation includes endoscopy or visual inspection with acetic acid. Other includes exploratory surgery/autopsy and radiologic studies. ICCC, International Classification of Childhood Cancer.

Pathology confirmation rates were high and varied across cancer sites: all sites combined, 67%; Hodgkin and non-Hodgkin lymphomas, 100%; breast cancer, 78%; and cervical cancer, 72% (Fig 3A). Kaposi sarcoma was primarily diagnosed clinically (59%); esophageal cancer was diagnosed primarily via endoscopy (52%) or pathology (41%). For diagnoses with pathology confirmation, the majority of esophageal cancers were squamous cell carcinoma (78%); adenocarcinoma was uncommon (6%). For breast cancer, invasive ductal carcinoma (65%) was predominant. For cervical cancer, the histology distribution was predominantly squamous cell carcinoma (90%) and adenocarcinoma (7%). Complete treatment information was generally unavailable for adult patients with cancer (64%); 24.0% had a record of chemotherapy, 2.5% received surgery, 8.0% received or were referred for palliation, < 1% received either hormonal treatment or radiotherapy, and 1% declined therapy.

### Children and Adolescents

**Fig 4:**
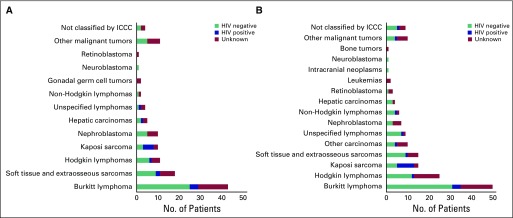
Distribution of patients with cancer and HIV status among children and adolescents: (A) female and (B) male. Non-Hodgkin lymphoma excludes Burkitt lymphoma. Leukemia includes lymphoid leukemias and other specified leukemias. Soft tissue and extraosseous sarcomas include rhabdomyosarcomas, fibrosarcomas, peripheral nerve sheath tumors, and other and unspecified soft tissue sarcomas, excluding Kaposi sarcoma. Other malignant tumors include other malignant epithelial neoplasms, malignant melanomas, and carcinomas. Not classified by International Classification of Childhood Cancer (ICCC) because of insufficient or absent histology information.

A total of 279 malignant tumors were recorded among pediatric and adolescent patients, representing 24% of patients in the registry. A diverse spectrum of childhood cancers was recorded. Burkitt lymphoma was the most common cancer diagnosed among girls (36%) and boys (34%; Figs 4A and 4B). Among girls, soft tissue and extraosseous sarcomas (rhabdomyosarcoma, fibrosarcoma, and other soft tissue sarcomas; 15%), Hodgkin lymphoma (9%), and Kaposi sarcoma (8%) were also common. Among boys, the most common cancers were also Hodgkin lymphoma (17%), Kaposi sarcoma (10%), and soft tissue sarcomas (10%). Overall, the majority of pediatric and adolescent patients received pathology confirmation of diagnosis (90%; Fig 3B). More than 96% of records for pediatric and adolescent patients had sufficient diagnostic information to be classified according to the International Classification of Childhood Cancer scheme.

HIV status was available for 63% of pediatric and adolescent patients (Figs 4A and 4B). Among children and adolescents, overall HIV prevalence was 18%, and prevalence was heterogeneous across cancer sites. HIV prevalence was low among patients with Burkitt lymphoma (8%). Among patients with Kaposi sarcoma, HIV prevalence was 61%. Information on receipt of antiretroviral therapy was generally not available among registry sources of information.

## DISCUSSION

Our study reports the contemporary burden of malignancies in a hospital-based cancer registry at KCH, a national teaching hospital in Lilongwe, Malawi. We demonstrated a large cancer burden of nearly 1,500 patients receiving care over a 21-month period. Our study describes an overview of the most commonly diagnosed cancers at the major public sector provider of oncology services in the northern and central regions of Malawi. Three notable features characterize the cancer caseload at KCH: a spectrum of cancer types that is strikingly different from high-income countries, young age at diagnosis, and high HIV prevalence.

Kaposi sarcoma and cancers of the cervix, breast, and esophagus were the most common types among women. Among men, Kaposi sarcoma, esophageal cancer, and non-Hodgkin lymphomas were most common. Our findings are consistent with regional data demonstrating that AIDS-defining malignancies are among the top 10 cancers in eastern and southern Africa.^1^ The prevalence of these cancers at KCH may also reflect oncology referrals and diagnostic services offered by a large tertiary care center. Visual inspection with acetic acid screening and screening for sexually transmitted infections at the sexually transmitted infection clinic may result in increased referrals for cervical cancer and other gynecologic malignancies. Similarly, referral for endoscopy services may drive the high prevalence of esophageal cancer observed in our hospital registry, although population-based estimates from 2007 to 2010 also showed esophageal cancer as the third most common cancer in Malawi.^3^ Of note, squamous cell carcinoma is the predominant histologic type of esophageal cancer in our hospital registry and the population-based registry,^3,14^ in contrast to resource-rich settings where adenocarcinoma is predominant.^15^

Children and adolescents comprised one quarter of patients in the registry. Burkitt lymphoma, soft tissue sarcomas, Hodgkin lymphomas, and Kaposi sarcoma were the most common pediatric malignancies, consistent with prior reports from Blantyre^16^ and other sub-Saharan African countries.^1^ We also observed a wide spectrum of rare pediatric tumors, including Wilms tumor (nephroblastoma), retinoblastoma, neuroblastoma, and bone tumors, as expected for a national teaching hospital.

Certain tumor types are likely to be underdiagnosed in our setting, in part because of limited or complete lack of diagnostic imaging via positron emission tomography, computed tomography, and magnetic resonance imaging. The low prevalence of urologic, GI, and visceral malignancies, including prostate, bladder, liver, pancreatic, stomach, colorectal, and lung cancers, in our registry is most likely a direct result of the scarcity of diagnostic imaging and difficulties with pathologic confirmation from visceral sites. Tumors of the CNS in children are also notably absent in our registry for possibly similar reasons.

Overall, the KCH cancer caseload is young: median age at diagnosis among adults is 47 years, and one quarter of all diagnoses for adults occurred between the ages of 20 and 36 years. Half of all adult patients with Kaposi sarcoma were younger than 40 years old. Strikingly, one quarter of patients with breast, cervical, and esophageal cancer were also diagnosed among patients younger than 40 years. As previously noted, referral patterns may be responsible for the striking age distribution of patients. However, young age at presentation for esophageal cancer has been similarly noted across Eastern Africa.^17^ Young population age structure overall in Malawi likely contributes in part to the observed age distribution of patients with cancer at our center.^18^

HIV coinfection is a significant comorbidity among adult and pediatric patients with cancer at KCH. HIV prevalence ranged from 22% among women with breast cancer to as high as 45% among those with cervical cancer, which is two to 3.5 times the prevalence of HIV among adult women in Malawi.^19^ HIV-positive Kaposi sarcoma was the leading cancer site among men and third most common site among women. The high burden of HIV-negative Kaposi sarcoma in our hospital is attributable to the high prevalence of the causal agent human herpesvirus-8 in southern Africa^20^ and likely represents endemic cases.^21,22^ HIV prevalence was 65% among men and women with non-Hodgkin lymphoma. For esophageal cancer, the second and third most common cancer among men and women at KCH, a more complete characterization of HIV prevalence is a priority. Although no known infectious agents have been associated with risk of esophageal cancer in Malawi,^23^ a case-control study in Zambia suggested a possible association with HIV.^24^

HIV status was more thoroughly documented among pediatric patients with cancer compared with adults in the registry. As expected, HIV prevalence was low among children with Burkitt lymphoma and is consistent with the HIV prevalence observed at the Queen Elizabeth Central Hospital in Blantyre.^25^ Pediatric Burkitt lymphoma is endemic to eastern Africa and is caused by Epstein-Barr virus,^26^ although case-control studies in Malawi and Uganda suggest a possible joint association with HIV.^25,27^ HIV prevalence among pediatric Kaposi sarcoma was substantially lower than the 89% prevalence reported in Blantyre,^28^ which highlights a need for continued vigilance of endemic pediatric patients, even during the modern HIV era in Malawi.

The design of the KCH Cancer Registry has several strengths. The registry is situated at one of two national teaching hospitals in the country, which is the sole provider of comprehensive oncology services in the central and northern regions of Malawi. In terms of efficiency, the registry was designed to systematically collect limited but essential subsets of data that are of direct relevance to our setting.^6^ A preliminary cost analysis of operations found a cost per case that was only slightly higher than the $10 to $15 estimate at well-established cancer registries in Kampala and Nairobi.^29^ Registry operations include active case finding across multiple data sources and departments, record consolidation, deduplication, and data quality and improvement activities, including periodic audits of completeness using laboratory records. The reporting system includes well-defined categories of suspected and verified cancer diagnoses,^11^ with sufficient granularity for research study planning and descriptive studies. An important facet of the design is collaboration with a high-volume pathology laboratory established in 2011, with whom the registry actively participates in data sharing of critical diagnostic information on patients with cancer.^8,30^ This is a major strength, given the paucity of pathology services throughout the region.^31^ Historic cancer surveillance in Malawi showed a pathology confirmation rate of 18%, with most cancer diagnoses supported by clinical, radiologic, and/or laboratory data.^3^ Low pathology confirmation rates in other population-based cancer registries from sub-Saharan Africa have been similarly noted.^32,33^ Only 4% of historic patients with cancer recorded by KCH from 2009 to 2010 had received histologic or cytologic confirmation through referral services to the Blantyre laboratory; the current rate of pathology confirmation is 65% for our hospital registry. In addition, we report for the first time a systematic, cross-sectional assessment of HIV prevalence among all patients with cancer at one of the largest oncology providers in Malawi. This is important because HIV is not a reportable disease in the region. Historically, the HIV status of < 1% of national registry patients was recorded.^3^ Conversely, the KCH Cancer Registry has contemporary information on HIV status for 45% of adult and 64% of pediatric and adolescent patients with cancer.

Our findings should be interpreted in the context of limitations from a hospital-based design. The wide geographic catchment area of the hospital and possible referral bias preclude estimation of cancer incidence using the KCH registry. As expected for a tertiary care center, one in five patients were referred from neighboring districts. Furthermore, the provision of specialized diagnostic services, such as visual inspection with acetic acid and endoscopy, and oncology treatment at KCH may be responsible for the over-representation of certain cancer types relative to the population-based registry. This is especially relevant for rare pediatric cancers for which treatment is generally unavailable outside of teaching hospitals in Malawi.

Limited information on stage at diagnosis and treatment arise from fragmented medical record keeping in a 2,000-bed hospital and other contextual constraints. Stage at diagnosis is seldom recorded in health records because of the lack of medical imaging, although late presentation is common in Malawi and other African countries. Further characterizing treatment needs, including surgery, chemotherapy, palliative care, and concurrent antiretroviral therapy, is a priority.^34-38^ We recognize that complete ascertainment of HIV status in our registry remains a challenge because of logistical and cultural challenges. Lastly, cancer survival and active follow-up for outcomes such as recurrence are not available through our hospital registry. Cancer survival, therefore, must rely on active tracing, the cost of which is prohibitive for routine, systematic cancer surveillance in Malawi at this time. However, we have also begun to address these issues through more detailed longitudinal clinical cohorts in high-burden cancers of interest.

Our findings provide an important overview of the contemporary cancer burden at an urban teaching hospital and major oncology center in Malawi. The findings of the KCH cancer registry demonstrate a high caseload among typically young patients and a significant burden of HIV infection among patients with cancer. The KCH Cancer Registry is an example of a low-cost, resource-efficient investment for local health care planning needs. In low- and middle-income countries with intermittent, sparse, or nonexistent cancer surveillance, hospital-based cancer registries can provide important local epidemiologic data while efforts to expand population-based registration continue.
